# Proteomics of Human Retinal Pigment Epithelium (RPE) Cells

**DOI:** 10.3390/proteomes6020022

**Published:** 2018-05-15

**Authors:** Sarka Beranova-Giorgianni, Francesco Giorgianni

**Affiliations:** Department of Pharmaceutical Sciences, University of Tennessee Health Science Center, Memphis, TN 38163, USA; sberanova@uthsc.edu

**Keywords:** retinal pigment epithelium, proteome, mass spectrometry, age-related macular degeneration

## Abstract

Retinal pigment epithelium (RPE) are specialized, multifunctional cells in the retina that form a monolayer of cuboidal, polarized cells adjoining the photoreceptor cells. The RPE are a critical component of the blood-retinal barrier, and they play essential functional roles for maintenance of retinal homeostasis and for support and health of photoreceptors. Age-dependent, progressive dysfunction and death of RPE cells and the resultant loss of photoreceptors contribute significantly to the development and progression of age-related macular degeneration (AMD) and other retinal degenerative diseases. Several different RPE cell culture models have been developed and utilized extensively as surrogates for cellular and molecular examinations of the RPE, and a large body of knowledge on RPE function in normal and pathological scenarios has been amassed in studies with cultured RPE. Proteomics has been an integral part of research efforts aimed to advance our understanding of RPE cell biology in health and disease. This review focuses on applications of proteomics to in vitro qualitative and quantitative investigation of human RPE cell culture models. The disease context discussed focuses on AMD.

## 1. Introduction

Despite the prevalence of visual disorders in the population worldwide, our understanding of the mechanisms underlying pathogenesis of many eye diseases remains incomplete. For close to two decades, proteomics has been an inherent component of research efforts in vision science aimed at improvement of mechanistic understanding of eye function in health and disease. In 2012, the Human Eye Proteome Project (EyeOme) was initiated as part of the Human Proteome Project [1] and, to date, proteomes in different compartments of the eye have been characterized in this endeavor [2,3]. This review focuses on the contribution of proteomics to our understanding of the biology of the retinal pigment epithelium (RPE) under physiological conditions and during the development of a common retinal degenerative disease of the elderly: age-related macular degeneration (AMD).

## 2. RPE Morphology and Function 

**Figure 1:**
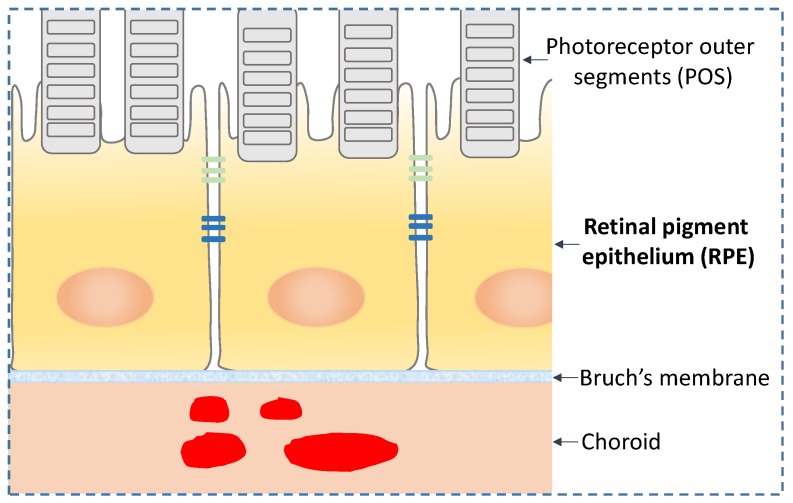
Location of retinal pigment epithelium (RPE) in vivo.

The retinal pigment epithelium (RPE) are polarized cells in the retina that form a monolayer at the interface between the photoreceptors and the extracellular matrix structure termed the Bruch’s membrane (BM) (Figure 1). At its apical side, the polarized RPE interact with the outer segments of the photoreceptors; at the basolateral side, the RPE receive and process input from systemic circulation via the choroid capillary system. The RPE are a critical constituent of the blood-retinal barrier, which enables selective transport of molecules in and out of the retina and preserves the immune privilege of the retina. The RPE are the cells with the highest phagocytosis activity in the human body. Through this activity, the RPE remove and recycle photoreceptor outer segment (POS) membranes and are therefore essential to maintain POS health. A thorough description of the RPE biology is outside the scope of this review; therefore, for a more in-depth knowledge on this subject, the reader is encouraged to consult the extensive body of literature available [4,5].

## 3. RPE and AMD

In the aging population, one of the most common and devastating retinal diseases is AMD, a multi-factorial, degenerative disorder of the central region of the retina (macula) caused by the death of the RPE, which is essential for the maintenance of the photoreceptor cells. AMD is the leading cause of legal blindness in adults > 60 years of age in the US and in other industrialized countries. According to the Centers for Disease Control and Prevention (CDC), over 1.8 million Americans 40 years of age and older are currently affected by AMD, and this number is expected to rise to 2.95 million by the year 2020 [6]. The impact of AMD on quality of life and economic losses is very significant [7]. Early stage AMD is not usually associated with significant vision loss, although signs of retinal dysfunction can be detected already at these stages. Early detection theoretically allows for early intervention, such as by nutritional means [8,9]. Despite the overall efficacy of these approaches, early stage AMD can still progress to advanced dry AMD, known as geographic atrophy (GA), or to exudative, neovascular (wet) AMD [10]. The highest risk for AMD is age [11]. In addition to age, immune dysfunction as demonstrated by the complement factor H (*CFH*) risk allele (Y402H) and environmental factors, in particular smoking, are also risk factors for AMD [12]. In recent years, numerous gene isoforms have been characterized by genome-wide association studies (GWAS) of large populations [13]. Regardless of the different genetic and/or environmental factors linked to AMD, a key, early manifestation of AMD is the formation of extracellular deposits known as drusen, which originate from cellular, lipidic and inflammatory waste material. Lipid-rich extracellular deposits accumulate in the Bruch’s membrane (BM) beneath the RPE throughout adulthood, increasing with age. The exact source of drusen have not yet been fully clarified although lesions, termed basal linear deposits (BlinD), formed within the inner collagenous layer of the BM are considered precursors of drusen [14]. Based on an extensive number of bioanalytical studies, it has been demonstrated that these lesions are largely composed of lipoprotein-like particles whose composition differs from lipoproteins found in systemic circulation. The BM lipoprotein deposits are comprised mainly of esterified cholesterol, triglycerides, unesterified cholesterol, phospholipids, and apoB 100, in proportions distinct from plasma lipoproteins [15]. Drusen composition also includes cellular debris, proteins and lipids many of which are oxidized [16,17]. The presence of myeloperoxidase [18] and iron [19] in the BM are potential factors for a highly oxidative milieu where accumulated proteins and lipids become oxidized. In fact, it is well documented that oxidized low-density lipoproteins (oxLDL) accumulate in drusen formations with aging [20].

## 4. In Vitro Models of RPE 

Extensive research, aimed at establishing in vitro biological systems amenable to study the molecular mechanisms of RPE biology and its alterations during AMD, has produced various cell models including different immortalized cell lines [21,22]. One of the most commonly used immortalized RPE cell lines is represented by the ARPE-19, which is a human derived retinal pigment epithelium cell developed from a 19-year-old male donor in the laboratory of Hjelmeland at the University of California, Davis [23]. The ARPE-19 cell line, under proper culture conditions, adopts the morphological molecular phenotypic characteristics of RPE cells in vivo, including polarization and development of proper barrier properties and expression of markers typical of native RPE cells [23,24]. Even though it is recognized that the ARPE-19 cell line might not fully display all the phenotypic features of native RPE cells, depending on cell passage and growth medium conditions [25], this cell line has been a reliable and valuable tool for cell biology studies related to RPE function in health and disease, as demonstrated by the extensive number of publications that have used this model—a PubMed search with the keyword “ARPE-19 cells” retrieved ca. 1300 publications between the year 2000 and 2018. Alternatives to cell line models to study RPE cell biology in vitro have emerged in recent years in particular human embryonic stem cell-derived RPE (hESC-RPE) and human induced pluripotent stem cell-derived RPE (hiPSC-RPE) [26]. Both cell models are considered better alternatives to ARPE-19 and other cell lines not only because they preserve most of the phenotypic characteristics of native RPE cells in vitro, but also because they have the potential to be used as cell transplants in patients affected by AMD. Primary RPE cells include fetal and adult cells. Fetal RPE cells are capable of proliferating in culture can be cryopreserved and developed to fully differentiated RPE cells [27]. Adult primary cells represent perhaps the closest cell model to the in vivo RPE. However, they have limited proliferation capability and therefore offer a limited experimental time window.

## 5. Proteomics of RPE 

One of the analytical approaches used to characterize native and derived RPE cells under physiological or altered conditions is represented by the characterization of the global protein content, i.e., the proteome, which represents one of the analytical strategies for the global-scale examination of the molecular composition of cells and other biological systems. Although gene expression analysis at the RNA level offers more coverage of least abundant transcripts, both transcriptomics and proteomics play a vital role in the understanding of complex biological systems. In general, there is a modest correlation between RNA transcripts and protein levels [28]. Furthermore, proteomics offers unique analytical capabilities not available to other omics methods. First, it is the only methodology that can identify and quantify in an unbiased manner protein post-translational modifications. Second, it can pinpoint subcellular location of target proteins. Third, it is the only method that can identify and quantify secreted proteins. Fourth, it can provide crucial data on specific protein-protein interaction networks. Major advances in mass spectrometry instrumentation and bioinformatics tools over the past ten years have dramatically transformed the field of proteomics. New types of high-resolution analyzers and analyzer configurations have been developed to enable fast data acquisition and high accuracy mass measurements [29,30]. These revolutionary technological innovations in mass spectrometry, together with advancements in chromatographic separation methods, have caused a departure from 2D gel electrophoresis-based strategies towards development and implementation of more powerful mass spectrometry-based workflows. With these workflows, it is now possible to identify and quantify thousands of proteins in cells, tissues and biological fluids. In this review, we highlight proteomics studies completed over the span of more than 15 years and their contribution to the advancement of our understanding of RPE cell biology in health and disease. We focus specifically on proteomics examinations of human RPE in vitro, including studies of primary RPE and of RPE cell culture models. We review investigations of the intracellular RPE proteome as well as analyses of the secretome and extracellular vesicle proteomes derived from RPE. In terms of disease context, we focus on proteomics studies in relationship to AMD.

### 5.1. Proteome Mapping 

In an early proteomics study focused on qualitative analysis of primary RPE cells, West et al. [31] generated the first human RPE proteome database. The RPE cells were isolated from normal adult human eyes; whole cell lysates as well as membrane, cytosolic, and microsomal fractions were prepared. Proteins were separated by 1D or 2D gel electrophoresis, and identified by matrix-assisted laser desorption/ionization time-of-flight (MALDI-TOF) MS and/or LC-MS/MS. With this bioanalytical strategy, a total of 278 RPE proteins were identified. This protein set was comprised of common housekeeping proteins as well as proteins involved in specialized functions of the RPE including visual cycle proteins and proteins associated with retinoid metabolism.

An important series of early proteomics investigations of the human RPE centered on lipofuscin. Lipofuscin are autofluorescent granules that accumulate in the RPE cells in association with aging, and that have been implicated in the pathogenesis of AMD. Lipofuscin is a complex mixture of different classes of biomolecules and encompasses bis-retinoids, chiefly *N*-retinylidene-*N*-retinylathanolamine (A2E), lipids and proteins, many of them oxidatively modified. Because of its connection to AMD and other retinal diseases, elucidation of the detailed molecular composition of lipofuscin to provide clues about the molecular basis of lipofuscin bioactivity has received a great deal of attention. Schutt et al. [32] completed the first map of the proteome in human lipofuscin. Lipofuscin samples were isolated from human RPE cells obtained from donors with no known ocular pathology. The analysis, conducted with 2D gel electrophoresis and MALDI-TOF-MS and LC-MS/MS, identified close to 80 proteins. Most of these proteins were abundant housekeeping proteins known to be present in RPE and in the photoreceptor cells. A follow-up study by the same group [33] extended the lipofuscin proteomics to characterize oxidative modifications that occur in lipofuscin proteins through lipid peroxidation and modification by advanced glycation end products (AGE). This investigation revealed that many lipofuscin proteins bear modification by AGE and/or lipid peroxidation products malondialdehyde (MDA) and 4-hydroxynonenal (HNE). The presence of oxidative modifications in human lipofuscin proteins was further confirmed by Warburton et al., who reported on the protein composition in lipofuscin [34] and melanolipofuscin [35], and who postulated that proteins, which are rendered non-degradable by oxidative modifications may at least in part account for lipofuscin accumulation in the RPE. Findings on the lipofuscin proteome were challenged in a report by Ng et al. [36]. In this study, lipofuscin was isolated from human RPE with a multi-step protocol that yielded highly purified lipofuscin granules. Analysis of this pure lipofuscin preparation showed only minimal protein content, thus suggesting that other classes of biomolecules and their oxidative modifications are mainly responsible for lipofuscin bioactivity.

### 5.2. Proteome Profiling: Intracellular 

Comparative studies of differentially expressed proteins in various in vitro models of RPE have been used to characterize the expression of markers characteristic of native RPE cells, in order to identify molecular mechanisms involved in cellular response to external stimuli and to identify the molecular pathways altered in diseased cells, e.g., AMD-derived RPE cells. 

Alge et al. [37] provided early insights in the differential protein expression profiles of primary differentiated RPE cells compared to dedifferentiated, proliferative RPE cells. In their study, the authors separated proteins by 2D gel electrophoresis and quantified them by protein spot imaging analysis. The differentially expressed proteins were identified by MALDI-TOF mass spectrometry. The study showed that proteins associated with specialized RPE functions, such as retinoid isomerohydrolase (RPE65) and cellular retinaldehyde-binding protein (CRALBP) were absent in the dedifferentiated cultured RPE cells. In contrast, proteins involved in cell shape, cell migration and proliferation were upregulated in the dedifferentiated cells. A subsequent study from the same group [38] expanded the comparative proteome examination of RPE cells to assess the proteome changes in differentiated primary human RPE as compared to the whole proteome of ARPE-19 and that of an immortalized RPE-derived cell line, specifically hTERT [21]. To differentiate between constitutively expressed proteins and de novo synthesized proteins, the cell culture procedure included incorporation of ^35^S-labelled methionine and cysteine. The extracted proteins were separated by 2D gel electrophoresis. Differentially expressed proteins spots were localized via computer-assisted image analysis of 2D gel spot patterns detected with total protein stain, as well as with autoradiography. The study found that in hTERT there was an upregulation of proteins involved in cellular migration, cell contraction, adhesion and migration, while proteins related to cell polarization were downregulated. The comparative analysis between the ARPE-19 and the primary cells was considered to be less conclusive in terms of functional groupings since the differentially expressed proteins belonged to functional clusters that were not interrelated. 

A comparative qualitative and quantitative proteomics analysis of human primary and embryonic stem cell-derived RPE (hESC-RPE), conducted by Hongisto et al. [39], identified a total of 1041 proteins commonly expressed, the majority of which were similarly regulated between the two cell models. These proteins included members of the visual cycle such as retinal-specific ATP-binding cassette transporter (ABCA4), retinoid isomerohydrolase (RPE65), retinaldehyde-binding protein 1 (RLBP1) and retinol dehydrogenase 11 (RDH11). Other relevant proteins commonly expressed in primary RPE cells also expressed in hESC-RPE were proteins known to play a role in extracellular matrix (ECM) and cell adhesion mechanisms, melanogenesis, phagocytosis and autophagy. This study demonstrated that hESC-RPE cells exhibit a proteome profile very similar to primary RPE cells.

A recent investigation focused on a comparative, quantitative examination of a proteome subset in cultured human fetal RPE cells versus the ARPE-19 cell line [40]. Specifically, this targeted proteomics study evaluated the expression of 41 plasma membrane proteins relevant for drug and nutrient transport, including the ATP-binding cassette (ABC) transporters and solute carrier (SLC) transporters. Absolute levels of these proteins in purified membrane fractions were determined with a quantitative targeted absolute proteomics method (QTAP) [41], which involved multiple-reaction monitoring (MRM) using stable isotope labeled peptide standards. Target peptides for quantification of each transporter protein of interest were selected in silico based on specific criteria including a unique amino acid sequence, a length of 6–16 amino acids, absence of methionine or cysteine, absence of post-translational modifications and a peptide sequence that is outside the transmembrane portion of the transporter protein. The study found that, out of 41 protein targets, 25 were below the limit of quantification in both cell models and the remaining protein transporters were expressed at similar levels between the ARPE-19 and the human fetal primary RPE cells.

Liao et al. [42] examined the proteome in ARPE-19 cells after repetitive exposure to tert-butylhydroperoxide to generate recurrent oxidative stress conditions that induced cell senescence in vitro. The workflow applied in this investigation included stable-isotope labeling of cysteine residues in the proteins with light or heavy acrylamide, followed by separation by ion exchange fast-protein liquid chromatography (FPLC) and subsequently by 2D gel electrophoresis. Proteins of interest were identified by MALDI-TOF MS. Relative quantification of the proteins in control vs. senescent cells was obtained from ratios of MS signal intensities of the peptide ion counterparts containing light or heavy label. The study uncovered increased levels in the senescent ARPE-19 cells of large fragments of three cytosolic enzymes glyceraldehyde 3-phosphate dehydrogenase (GAPDH), triosephosphate isomerase, and M2 pyruvate kinase. These findings suggest that RPE senescence is associated with perturbations of protein degradation mechanisms and resulting accumulation of protein debris material within the RPE.

Arnouk et al. [43] utilized the D407 human RPE cell line [22], together with primary bovine RPE, to examine the effects of oxidative stress on the RPE proteome. Cells in culture were treated with non-lethal dose of H_2_O_2_ to induce oxidative stress. Alterations in protein expression were investigated with a difference gel electrophoresis (DIGE) approach that uses protein labeling with spectrally resolvable CyDyes to enable multiplexed separation and protein quantification of different samples in the same 2D gel. Differentially expressed proteins in spots revealed by DIGE were identified by MALDI-TOF and MALDI-TOF-TOF mass spectrometry. The proteome in H_2_O_2_-treated D407 cells showed upregulation of several proteins including heat shock proteins, peroxiredoxins, elongation factor Tu, and RNA helicase DDX3X. Findings from this study were expanded in subsequent investigations that applied global and targeted proteomics approaches in the context of interactomics and phosphoproteomics [44,45,46]. 

Oxidative stress-related lipid and protein modifications have long been implicated in the etiology of AMD. Glenn et al. [47] assessed the alterations in the proteome of ARPE-19 exposed to a Bruch’s membrane (BM) substrate that had been oxidatively modified with advanced glycation end products (AGE). The study involved the use of 2D gel electrophoresis for protein separation, gel image analysis and LC-MS/MS for identification of differentially expressed proteins. Among the differentially expressed proteins, the authors highlighted the deubiquitinating enzyme ubiquitin carboxyl-terminal hydrolase isozyme L1 (UCH-L1), which was upregulated in AGE-exposed RPE cells. This finding suggests that oxidative stress-related modifications in the BM may cause disruption of the protein degradation function in the RPE cells, which results in accumulation of damaged proteins and may lead to RPE cell death.

Apart from age, the strongest environmental risk factor for AMD is cigarette smoking. Smoke-induced cellular damage is mediated through induction of oxidative stress, depletion of antioxidants, and complement activation [48,49]. A comparative proteomics study reported by Merl-Pham et al. [50] focused on identification of proteome alterations in ARPE-19 cells treated with a sub-lethal dose of cigarette smoke extract (CSE). Using label-free quantification with LC-MS/MS, the investigation profiled close to 2000 proteins based on quantification of at least two unique peptides, and revealed that 184 of the profiled proteins exhibited ≥2-fold expression changes upon CSE treatment. Pathway enrichment analysis with this set of differentially expressed proteins indicated CSE treatment affected a number of different biological processes, chiefly RNA processing, RNA transport, and extracellular matrix remodeling.

Nordgaard et al. conducted an extensive proteomics analysis of primary RPE cells obtained from human donors affected by AMD [51]. The RPE proteome of four progressive stages of AMD were analyzed and compared. Whole proteome extracts were separated by 2D gel electrophoresis followed by gel image analysis to pinpoint protein spots that displayed differences in intensity between the four disease stages. The differentially expressed proteins were identified by MALDI TOF MS and by LC-MS/MS. This study identified changes in protein expression levels in the early disease stage that included heat shock proteins and components of apoptotic signaling pathways, such as αA crystallin, voltage-dependent anion-selective channel protein 1 (VDAC1) and glutathione S-transferase pi (GST-π). Some of the identified changed proteins were associated with mitochondrial function. Late stage AMD-derived RPE cells exhibited protein expression changes for two retinoid binding proteins: CRABP1 and CRALBP. These results suggest that late AMD stage protein changes are associated with perturbation of retinoid-related functions. In a follow-up study on protein level changes in RPE isolated from different stages of AMD, Nordgaard et al. focused exclusively on the mitochondrial proteome [52]. The study used the same methodological strategy as described in the previous investigation and identified eight differentially expressed mitochondrial proteins. Interestingly, mitochondrial elongation factor Tu (Tufm) was found to be overexpressed in early AMD stage RPE cells. Tufm has been shown to be an essential mitochondrial translation and elongation factor. Overexpression of Tufm in vitro can rescue mitochondrial protein expression in fibroblasts derived from patients with a mitochondrial protein expression deficiency [53]. This finding suggests that overexpression of Tufm may alleviate mtDNA damage in early AMD development. A second finding of this study relates to a decreased expression level of several subunits of the ATP synthase complex in RPE cells from AMD stages 3 and 4, which suggests that decreased levels of intracellular ATP could impact cell function in late stage AMD. Finally, mtHsp70 expression was found to be downregulated in late stage AMD samples. Mitochondrial Hsp70 is involved in transport of nuclear-encoded proteins into the mitochondria. Downregulation of mtHsp70 may result in a functional impairment of various mitochondrial protein modules essential for cell metabolism.

Murad et al. studied the role of miR184 in human RPE cells and its relevance to AMD [54]. Quantitative proteomics with iTRAQ labeling, multi-dimensional chromatography and tandem mass spectrometry was used to identify proteins regulated by miR184 in RPE cells transfected with a miR184 inhibitor. The analysis revealed ezrin, a protein required for the formation of microvilli and membrane ruffles in epithelial cells, as the protein with the highest increase in expression following miR184 inhibition. Subsequent cell and molecular biology investigations allowed to postulate a mechanistic molecular link between miR184 and phagocytosis in RPE cells, suggesting an important role for this miRNA in AMD.

A proteomics study by Yang et al. [55] of induced pluripotent stem cells-derived RPE (iPSC-RPE) from AMD patients with high-risk, single-nucleotide polymorphism (SNP) at the ARMS2/HTRA1 locus and non-AMD controls demonstrated that in vitro treatment with the bis-retinoid A2E, a major component of lipofuscin, and exposure to blue light elicits a large increase in protein expression of the anti-oxidant superoxide dismutase 2 (SOD2) enzyme only in iPSC-RPE cells derived from non-AMD controls. The authors postulated that the diminished SOD2 protective response observed in the high-risk ARMS2/HTRA1 genotype causes an increase in the protein ratio between phosphorylated FOXO3A and non-phosphorylated FOXO3A transcription factor, which in turn causes a decrease in the level of β-catenin, an adherence junction protein essential to maintain the RPE cell-cell junctions. 

### 5.3. Proteome Profiling: Extracellular

In recent years, an increased focus has been directed to global-scale examination of proteins secreted by cells into the extracellular space, i.e., secretome. The secretome includes soluble proteins as well as proteins secreted as part of extracellular vesicles. These proteins are of great biological interest because they can potentially function as mediators of cell-to-cell signaling and can therefore modulate the behavior of cells that can be proximal or distantly located. Furthermore, these secreted proteins could be used as disease biomarkers. 

An et al. [56] focused on global-scale survey of proteins secreted from RPE cells to identify proteins potentially relevant for molecular mechanisms of AMD, in particular in the context of drusen formation. The secretome of cultured primary RPE cells harvested from donors with AMD and from age-matched unaffected controls was profiled. The RPE cells were genotyped for the Y402H complement factor H (*CFH*) variant that is associated with increased risk of AMD [57,58]. The bioanalytical workflow encompassed stable-isotope labeling in culture (SILAC), separation of secretome proteins by SDS-PAGE, and identification and quantification of the proteins by LC-MS/MS on a linear ion trap mass spectrometer (LTQ). A total of 72 proteins from diverse functional categories were identified in the RPE secretome, including cell adhesion/connective tissue proteins, complement factors, proteases and protease inhibitors. Among the secreted proteins that were differentially regulated in the RPE secretome from AMD vs. control were known components of drusen, supporting a role of the RPE in drusen biogenesis.

In a later study, An et al. [59] examined the effect of the high temperature requirement 1 (HTRA1) protease on the RPE secretome. In this study, primary RPE cell cultures were established from donors possessing a *HTRA1* promoter variant allele associated with increased AMD risk, as well as from donors with normal (wild-type) genotypes. The bioanalytical strategy incorporated SILAC in combination with SDS-PAGE for protein separation, and LC-MS/MS on an ion trap-Orbitrap mass spectrometer for protein identification and quantification. Compared to wild-type cells, RPE cells homozygous for the *HTRA1* risk allele expressed higher levels of the protease, and they also showed a 2-fold increase in HTRA1 secretion. To pinpoint specific protein targets of HTRA1 in the RPE secretome, proteins secreted in the conditioned media were treated with recombinant HTRA1. Out of a total of 196 proteins identified in the RPE secretome, eight proteins were found to be selectively cleaved by HTRA1, as measured by a decrease of their abundance in the secretome. These HTRA1 substrates encompassed proteins involved in the complement cascade and in regulation of amyloid deposition, the latter suggesting a link between *HTRA1* AMD risk genotype and formation of drusen deposits.

Alcazar et al. [60] investigated the proteome in blebs formed by RPE upon exposure to non-lethal dose of hydroquinone (HQ), an environmentally relevant compound that is also a major component of cigarette smoke [61]. Blebs are microvesicles of range of sizes that form by direct budding and pinching off the plasma membrane. Blebs were isolated from conditioned media of HQ-treated ARPE-19 cell cultures by low-speed centrifugation, the proteins were separated by SDS-PAGE, and identified by LC-MS/MS. A total of 314 proteins were identified with this bioanalytical strategy, including a number of proteins involved in cell adhesion and immune response. One of the findings highlighted in the study relates to the presence in the blebs of a glycosylated form of basigin, a transmembrane protein known to modulate extracellular matrix metalloproteinases (MMP). Incubation of naïve ARPE-19 cells with bleb-containing conditioned medium showed an increased activity of MMP-2, a protein which plays a role in extracellular matrix remodeling in the retina. This result indicated that blebs generated by HQ-treated RPE contained bioactive cargo that may participate in remodeling of the extracellular matrix. In more general terms, this finding supported the postulation that blebs produced by the RPE may serve as vehicles for delivery of bioactive molecules to modulate cell function at different sites in the retinal microenvironment, with possible implications in AMD pathogenesis.

The proteome of another type of extracellular vesicles secreted by the RPE—the exosomes—was the focus of a research study reported by Biasutto et al. [62]. Exosomes are small 30–150 nm membrane-enclosed vesicles that originate intracellularly via multivesicular bodies that eventually release exosomes through fusion with the plasma membrane [63]. The study employed the ARPE-19 cell model and probed the exosomal proteins and phosphoproteins, with particular emphasis on changes in the phosphoproteins resulting from non-lethal oxidative stress induced in the parent RPE. Exosomes were isolated from conditioned media by differential ultracentrifugation, and the constituent (phospho)proteins were profiled using a multiplexed immunoassay-based approach with a reversed phase protein microarray (RPMA) platform [64]. Seventy-two proteins, including 41 phosphoproteins, were detected in the RPE exosomes by RPMA analysis. Abundance levels of many exosomal (phospho)proteins were different from levels of these proteins in parent cell lysates, indicating that a specific protein cargo is selectively packaged and secreted by the RPE. Importantly, a number of the probed phosphoproteins also exhibited different abundance levels in exosomes derived from control vs. stressed cells. These results indicated that the parent RPE secrete condition-specific cargo containing elements of phosphorylation-dependent signaling pathways to modulate signaling, and hence function, of other (recipient) cells. Finally, the authors hypothesized that exosomes secreted by the RPE may be a source of phosphoproteins detected in vitreous humor.

Kang et al. [65] examined whole secretome and exosomal proteome of the RPE as potential sources of protein biomarkers in aqueous humor (AH) of AMD patients. Exosomes isolated from AH of AMD patients and of unaffected controls were investigated. To elucidate the contribution of RPE-specific exosomes to the exosome content of AH, ARPE-19 cell cultures were used to produce conditioned media for secretome analysis, and for exosome isolation and analysis. To induce oxidative stress, ARPE-19 cells were treated with paraquat. Exosomes were obtained from conditioned cell culture media or AH by precipitation using a commercial exosome isolation kit. Furthermore, the bioanalytical workflow utilized SDS-PAGE for protein separation and LC-MS/MS for protein identification. Selected exosomal proteins in AH samples were quantified by MRM. The comparative proteome study indicated RPE as a potential major source of exosomes present in AH. Several proteins, including cathepsin D and cytokeratin 8, were identified as putative AMD biomarkers in AH.

## 6. Conclusions

Delicate balance in the retinal microenvironment requires complex communication and interplay among different cell types to respond to environmental challenges and to maintain homeostasis. Development of AMD is connected to perturbations of this balance. The RPE are responsible for the support of the photoreceptors and hence are critical for vision. Progressive dysfunction and death of RPE cells and the resultant loss of photoreceptors are causally linked to the development and progression of AMD. Therefore, elucidation of the mechanisms underlying RPE death is necessary to drive the design of new and improved therapies. To this end, modern omics technologies can provide the mechanistic insights necessary to elucidate the biological pathways relevant to disease onset and progression. Thanks to major technological advancements in instrumentation hardware and software, proteomics has emerged as a leading research area that can provide new information related to the perturbations in intracellular protein composition and quantity that characterize the phenotype of RPE cells in different stages of AMD development. In addition, opportunities are ripe for proteomics applications to be further expanded to characterize and quantify RPE-derived extracellular vesicles. As in all major life science disciplines, there has been a time lag between proteomics-based discoveries and their translation to disease treatment; nevertheless, both areas of investigation are uniquely poised to make seminal contributions to the understanding of the molecular mechanisms that underlie a multi-factorial, complex disease such as AMD.
